# Willingness and Barriers to Voluntary Counselling and Testing Among Self-Perceived Healthy Adults in Tanzania

**DOI:** 10.24248/EAHRJ-D-18-00020

**Published:** 2019-07-30

**Authors:** Michael A Munga, Janesta A Urassa, William J Kisoka, Prince P Mutalemwa

**Affiliations:** a National Institute for Medical Research, Dar es Salaam, Tanzania; b Ardhi University, Dar es Salaam, Tanzania

## Abstract

**Background:**

Despite the ongoing efforts to promote HIV testing, the majority of adults in Tanzania remain untested, and many remain unwilling to know their HIV status. Understanding the underlying reasons for this unwillingness to test and know one's status will support the development of targeted interventions to promote the uptake of HIV testing. This paper explores the willingness of and barriers faced by self-perceived healthy individuals to test for HIV in selected districts of Tanzania.

**Methods:**

A cross-sectional survey was conducted in urban and rural wards between October 2011 and March 2012. Structured questionnaires with closed- and open-ended questions were administered to heads of randomly selected households. Information collected included socioeconomic, demographics, rural/urban backgrounds and the perceived reasons which hinder household heads/members to access and utilise HIV-testing services. Regression analysis was conducted to assess the relationship between the same factors and participants' willingness to go for an HIV test in the near future.

**Results:**

There were 1,429 respondents from randomly selected households interviewed, and out of these, 57.1% were women, and 42.9% were men. The mean age of all respondents was 33.6 years; men were slightly older (mean age, 37 years) than women (mean age, 34 years). Almost one-third (n=433, 30.3%) of the respondents reported having ever tested for HIV, of whom 294 (61.8%) were women, and 139 (38.2%) were men. Being educated to at least the primary school level, being an urban resident, and being female increased the probability of HIV testing by 1.7% (*P*<.001), 1.3% (*P*<.005) and 0.2% (*P*<.005) respectively. Further, for each year, one's age increased the probability of positive future intentions to test for HIV increased by 0.4 % (*P*<.005). Education, residence and marital status were not significantly associated with future willingness to test. Fear of being stigmatised and discriminated was observed to be one of the important barriers for HIV testing among those who had never tested and those who were unwilling to test in the future.

**Conclusion:**

In urban areas, knowledge of the benefits of HIV testing is higher than in rural areas. Overall stigma remains the most salient barrier to HIV testing and interventions that address this, and other structural drivers for stigma need to be addressed in order for people's willingness to test to increase. Finally, health systems need to be strengthened to further encourage testing and be ready to provide quality and non-discriminatory services once people's willingness to test becomes apparent.

## INTRODUCTION

In December 2013 the UNAIDS set the 90-90-90 targets, which called for 90% of people to know their status, among whom 90% should be linked to care, among whom 90% should be virally suppressed.^1^ Despite high and improved knowledge about HIV across the country,^2^ in Tanzania the first “90” remains a bottleneck. The majority of adults in the United Republic of Tanzania remain either untested or unwilling to know their HIV status.^3^ Reasons for this pattern had not been systematically and comprehensively established in the Tanzanian literature.

The number of voluntary counselling and testing (VCT) sites in the country has rapidly expanded to 2,137.^4^ According to the 2010-2011 Malaria and AIDS Indicator Survey, more than 90% of people knew where to get an HIV test.^4^ In 2013, Tanzania introduced new HIV testing approaches such as home-based testing and community testing.^4^ Provider-initiated testing and counselling (PITC), wherein a health-care provider specifically recommends an HIV test to someone attending a health facility and performs the test unless the patient declines, has also been introduced.^5,6^ Data from the THIS 2016-2017 indicates that 67% of women and 50% of men had been tested for HIV at least once.^2,5,6^ While this represents an increase from the 2013 rates (Tanzania's UNAIDS 2014 progress report found in 2013 only 28.4% of people aged 15-49 had taken an HIV test in the past 12 months and knew their results^5^) it still falls short from UNAIDS 90-90-90 target.

**FIGURE:**
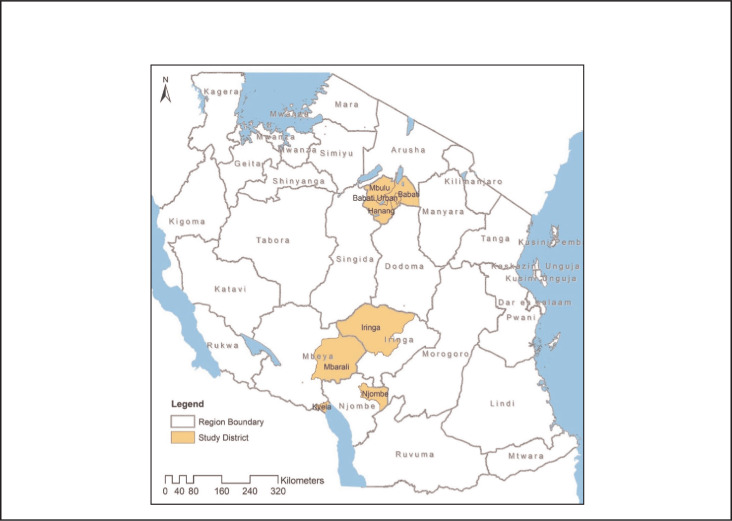
Map of Tanzania Highlighting the Study Districts

In 2016, Tanzania adopted a policy of universal test and treat (UTT) for the general population. Under UTT, all who test HIV positive will be initiated onto antiretroviral therapy (ART) regardless of their immune status. Translated into practice, this means that adults who test positive could still feel healthy when initiated on treatment. Evidence for the benefits of UTT policies to reduce onward HIV transmission is growing. The recent PopART trial suggests that if the 90-90-90 targets are reached, population transmission of HIV can be reduced by up to 30%.

Considerable efforts are needed if Tanzania is to reach the first “90” target. This study aimed to identify and analyse the willingness and barriers to VCT among self-perceived healthy adults. There has been significant documentation of the barriers to VCT in Tanzania; however, little has been documented since the widespread implementation of UTT. Understanding the drivers to VCT uptake in this context will be important to better direct efforts towards improve testing.

## METHODS

### Study Design and Setting

A cross-sectional study was conducted between October 2011 and March 2012. A structured questionnaire was administered in Swahili to heads of randomly selected households. The study was conducted in 7 districts: Mbulu, Babati rural, Hanang, Njombe, Iringa rural, Kyela, Mbarali (see table 1).

**TABLE 1.** Characteristics of Study Districts

District	Region	Regional HIV prevalence	Population	Main Economic Activities
Mbulu	Manyara	1%	237,882	Farming, livestock keeping, fishing, and petty trading
Babati Rural District	Manyara	1%	303,013	Farming, livestock keeping, fishing, and petty trading
Hanang	Manyara	1%	275,990	Farming, livestock keeping, petty trading, and bee keeping
Njombe	Formally Iringa, now Njombe region	>10%	130,223	Farming, livestock keeping, fishing, petty trading, logging and timber production
Iringa Rural	Iringa	>10%	245,623	Farming, livestock keeping, fishing, petty trading, logging and timber production
Kyela	Mbeya	>10%	~200,000	Fishing, livestock keeping, and petty trading
Mbarali	Mbeya	>10%	234,908	Farming, livestock keeping, fruit processing

### Sampling Strategy and Sample Size

A mix of purposive and random sampling was conducted. A multistage sampling strategy was adopted. The 2 highest prevalence regions in the country (Iringa and Mbeya) were purposively selected, and the final region (Manyara) was randomly sampled from the remaining 24 administrative regions recognised at the time of the study period. Rural-urban stratification was done in consultation with district authorities in each selected district. Two wards were selected from each district (1 ward was classified as rural and another urban). From each ward, 1 village was selected, making 2 villages from each district. A Probability Proportional to Size (PPS) approach was used to estimate the required number of household from each village. We first calculated the proportion of the village population to the total population of the district. We then used the calculated proportions to establish the final sample from the village population. A random sampling technique was finally used to select households to meet the calculated sample size in each village.

### Household Survey

A structured pretested questionnaire was administered face-to-face in Swahili to male or female heads of households by the trained research assistants who were supervised by 2 researchers in each study district. The interviews were managed by research assistants, and each interview took an average of between 45 minutes and 1 hour and took place in the respective participants' households or an area chosen by them. The tool was first pilot-tested to assess whether it was capable of capturing the required information, whether the questions asked to the respondents were comprehensively understood and whether the research assistants consistently and uniformly asked the questions in a way that was easy for respondents to comprehend logically. All the observed problems related to the content and structure of the questions, logic and consistency were considered and addressed during the tool refinement workshop which involved both researchers and research assistants and took place in November 2011. The survey tool collected data on socioeconomic, demographics, rural/urban backgrounds and the perceived reasons that hinder or help household members to access and use VCT services. Willingness to go for VCT in the near future (any time between day 1 and the subsequent 90 days after the date of interview) was asked. Participants were asked to rate their health state on a scale of ‘0’ to ‘100’; whereby ‘0’ denoted poor health and any number between ‘50’and ‘100’ denoted good health. Those who self-rated between ‘50’ and a ‘100’ were considered healthy and included in the study. Those who self-rated below ‘50’ were excluded from the study.

### Data Analysis

Data were double entered into a computer database using Epidata^®^. Responses from open-ended questions were post-coded before being entry. Data quality checking and analysis were performed using Stata software (StataCorp, College Station, TX, USA). Chi-square tests and tests of association (regression analyses) were performed to ascertain the association between independent and dependent variables. In order to perform the regression analysis, numerical variables were transformed to become categorical variables.

**TABLE 2.** Association Between Respondents' Characteristics and Willingness to Attend Voluntary Counselling and Testing in the Future (Study Participants Who Did Not Test, N=996)

Variable	Coefficient	*P* Value	95% Confidence Interval	
**Gender (being a woman)**	0.0187511	.992	−2.88	3.00
**Education**	0.2813292	.670	−1.01	1.58
**Marital status (being married)**	-0.8562862	.572	−3.82	2.11
**Age**	0.0466927	.050	0.062	0.10
**Residence**	0.0041625	.856	0.96	1.05
**Cons_**	0.0706359	.892	−1.09	0.95

### Ethical Considerations

Ethical clearance was obtained from the Medical Research Coordination Committee (MRCC) of the National Institute for Medical Research (NIMR). The protocol was subjected to scientific and ethics review process, approved and given certificate number NIMR/HQ/R.8a/Vol.IX/1112. In addition, study permission was sought from the respective authorities from whom our potential participants were recruited. Both verbal and written consent were sought and obtained from participants.

## RESULTS

### Characteristics of the Study Participants

The study involved 1,429 respondents from randomly selected households in urban (49.6%) and rural (50.4%) wards, and 57.1% were women. The mean age of all respondents was 33.6 years. On average, men were slightly older (mean age, 37 years) than women (mean age, 34 years). The majority (62.9%) of respondents had completed primary school. Only 13.6% reported having completed ordinary level secondary education. Less than 2% had postsecondary education. The majority (67.3%) of the respondents were married; 17.8% were single, and the remaining 14.9% were either cohabitating or divorced.

**TABLE 3.** Association Between Respondents' Characteristics and Opting for Voluntary Counselling and Testing (N=1,429)

Variable	Coefficient	*P* Value	95% Confidence Interval	
**Gender (being a woman)**	0.0180538	.05	0.0058	0.30
**Education**	0.0172017	.05	0.0035	0.20
**Marital status (being married)**	0.1003441	.72	-0.45	0.65
**Age**	0.0482593	.66	-0.13	0.48
**Residence**	0.01318902	.05	0.0054	0.37
**Cons_**	2.9484361	<.001	1.62	5.49

### Use of VCT Services

Overall, 433 (30.3%) of the respondents reported having undergone an HIV test. When disaggregated by area of residence, 57.3% of respondents from urban areas said they have ever tested for HIV compared to 42.7% of rural respondents. More women reported having ever been tested for HIV (36.05%) than (25.9%) of men. Female sex was significantly associated with an increased probability of testing (*P*<.005). This pattern was consistent even after adjusting responses for place of residence. The majority of participants had primary school-level education. Being educated, at least up to primary school level, significantly increased the probability that a person will test for HIV by 1.7% (*P*<.001). A point increase in age was significantly associated with the probability of HIV-testing by 0.4 % (*P*<.005).

After accounting for the requirement for testing recommended under PMTCT, the majority of participants (72.6%) reported that the main motivation for VCT was a self-perceived risk of transmission, which participants defined as having sexual intercourse with partners who were perceived as being involved in high-risk sexual activity.

### Reasons for Not Seeking VCT Services

The majority of respondents (n=996, 69.7%) across all study districts had never sought VCT services at the time of the study, despite the ongoing campaigns emphasising the importance of testing. The majority (mainly men) of respondents who confessed not to have sought VCT services pointed 4 reasons as main drivers for not going for VCT namely: distance from VCT centres associated with travel costs, HIV/AIDS-related stigma and discrimination, fear of people seeing them entering a VCT centre (stigma) and the knowledge that there is no cure for HIV (Table 4).

**TABLE 4.** Reasons and Motivations for Attending Voluntary Counselling and Testing Among Participants Who Tested (N=433)

	Men n (%)	Women n (%)	*P Value*
**Reason/Motivation**			
Self perception of high-risk sex behaviour	314 (72.6%)	119 (27.4%)	.05
Sexual relationship to a partner-with high risk sex behaviour	291 (67.3%)	142 (32.7%)	.05
Frequent illnesses	220 (50.9%)	213 (49.1%)	.06
Recommendation from a sex partner	313 (72.3%)	120 (27.7%)	.05
Self reassurance of negative HIV status	130 (30.1%)	303 (69.9%)	.05
Compulsory PMTCT for pregnant women	267 (68.5%)	136 (31.5%)	.05
**Knowledge indicator**			
Promotes behaviour change (to avoiding risky behaviours)	237 (54.8%)	196 (45.2%)	.07
Promotes the Prevention of Mother to- Child Transmission (PMTCT) of HIV/AIDS	153 (35.4%)	280 (64.6%)	.005
Poses as an entry point for treatment programs for STIs	142 (32.8%)	291 (67.2%)	.05
Poses as an entry point for diagnosis and treatment of tuberculosis/HIV co-Infections	209 (48.2%)	244 (51.8%)	.07
Enhances timely initiation of ARVs to HIV/AIDS patients	125 (28.9%)	308 (71.1%)	.05
Provides knowledge that reduce stigma and discrimination to infected people	138 (31.9%)	295 (68.1%)	.05

### Future Willingness to Seek VCT Services

Increasing age was the only factor significantly associated with positive future intentions of testing for HIV (*P*<.005) (Table 3). Gender, education, residence and marital status were not significantly associated with positive future intentions to test.

### Barriers to VCT Among Those Who Had Not Tested

Of the 69.7% of participants who said they never attended VCT (n=996); fear of stigma and the associated discrimination were the most cited barriers. Men were more fearful than women to attend services (*P*<.05). Another important barrier for those who did not test for HIV was that testing centres were too far from where they live (n=349, 30.5%), incurring travel costs (n=199, 20%) and a perceived fear of insufficient confidentiality at VCT sites.

### Knowledge of VCT Benefits Among Those Who Tested

Respondents were asked about their understanding of the benefits of VCT (Table 5). Women were more knowledgeable than men. More than half (57%) of all women respondents who had ever gone for VCT services said that they knew that VCT promotes behavioural change, especially in relation to avoiding risky sex. The level of knowledge that VCT helps as an entry point for the diagnosis and treatment of HIV/AIDS and tuberculosis coinfections was generally higher for women than men. In addition, more women who had ever gone for VCT services were aware that higher levels of VCT uptake could help to reduce HIV/AIDS-related stigma and discrimination. They were additionally more aware of the role that VCT has in building confidence among the PLWHA. Adjusted for area of residence (Table 5), urban residents were more knowledgeable about the benefits of VCT than rural participants.

**TABLE 5.** Knowledge of Voluntary Counselling and Testing Benefits Among Participants Who Tested for HIV Adjusted for Area of Residence (N=433)

Knowledge Indicator	Urban n (%)	Rural n (%)	*P* Value
**Promotes behaviour change (to avoiding risky behaviours)**	245 (61.3%)	168 (38.7%)	.05
**Promotes the Prevention of Mother to- Child Transmission (PMTCT) of HIV/AIDS**	(58.8%)	(41.2%)	.05
**Poses as an entry point for treatment programs for STIs**	265 (61.2%)	168 (38.8%)	.05
**Poses as an entry point for diagnosis and treatment of tuberculosis/HIV co-Infections**	222 (51.2%)	211 (48.8%)	.06
**Enhances timely initiation of ARVs to HIV/AIDS patients**	283 (65.3%)	3150 (4.7%)	.05
**Provides knowledge that reduce stigma and discrimination to infected people**	217 (50.1%)	216 (49.9%)	.06

## DISCUSSION

This study analysed VCT utilisation among self-perceived healthy adults in 7 districts of Tanzania, including from 2 of the highest prevalence regions in the country. The study provides a descriptive analysis of the intentions, motivations and barriers to VCT among people in urban and rural settings in Tanzania.

Overall, 30.3% of all those interviewed reported having had ever tested for HIV. This proportion falls far short of the UNAIDS target for 90% of people to know their HIV status. Studies have shown that in many developing countries, VCT services are vastly underutilised despite the growing knowledge on the benefits of testing.^2,7^ Our study suggests that being an urban resident is an important predictor for HIV testing. Typical developing country settings such as Tanzania, have a skewed distribution of many social services (including health services and VCT service centres) to the disadvantage of rural residents.^3,8-10^ That is, not only that VCT and its associated services such as availability of IEC materials are concentrated in urban areas but also the infrastructure and the health workforce needed to offer these services are more developed in urban areas than in rural areas. In our study, we did see a higher proportion of VCT users in urban areas than in rural areas. Moreover, our findings point out that more women reported having ever tested for HIV, most likely influenced by the widespread availability of and contact with antenatal HIV counselling and testing among women.

Respondents provided various reasons for their attitudes towards VCT. The majority were willing to use the service as they just wanted to be sure of their HIV/AIDS status. For others, their self-perceived risk of HIV infection as a result of practising high-risk sexual behaviour was a motivating factor for seeking VCT services. These findings confirm what has been reported elsewhere,^2,7,9-17^ namely that uptake of VCT services is highly influenced by an individuals' perception of risk of being infected. Similar observations have been reported elsewhere.^4-6,10,12,18^ Those at highest risk of infection should be those most targeted to take up VCT services; our findings suggest that those who self-identify as being at high risk are more likely to access services. However, the question remains, do all at risk, self-identify as being at high risk? Further research is needed to explore risk perceptions and consider how they may also change over time.

Our study illustrated that knowledge of VCT benefits is higher among those who had previously tested than those who had not. Further, women were more knowledgeable than men. It is likely that the testing that forms part of ANC services improves knowledge. Findings from other studies have demonstrated that a high level of knowledge does not necessarily lead to a higher uptake of VCT services.^2,3,7-10,12,14-17,19^ Therefore, while knowledge is important for decision making, other factors are more critical in influencing a person's behaviour and subsequent uptake of VCT services.

Our study findings suggest that the majority of those who had not ever tested were willing to do so in the future. We found that increases in age led to an increased probability for future willingness to go for VCT. This might be explained by the fact that as people grow older, they become more concerned with their health; this needs to be further explored. Our study found that some respondents had no future intention to test for HIV. There are many factors which might have influenced their likelihood of not testing, for many these same factors would have also acted as barriers to previous testing. Confusion and desperation among those who had never tested is a critical issue which may make people unresponsive to VCT services, even after being informed of the benefits of doing so. In our study, some of those who had negative future willingness to attend VCT claimed that “just knowing that there is no cure for HIV/AIDS is enough reason for not seeking voluntary counselling and testing services”.^7,14-17,20,21^ Policies, programmes and interventions aimed at increasing VCT uptake should focus on the structural factors which prevent potential clients from seeking VCT services; these include stigma and the associated discrimination, poverty (manifesting as an inability to afford the costs of travel). Additionally, findings from this study indicate that several health systems barriers exist. Many respondents mentioned that there were shortages of qualified health workers, a lack of confidentiality associated with health workers who do not abide to ethics because of poor training they received prior to assuming counselling and testing responsibilities, and poor health facility infrastructure which did not permit sufficient confidentiality especially in areas where the level of stigma and discrimination against PLWHA is high.^16,17,20,21^

Our findings resonate with many others to clearly indicate that stigma remains a critical factor preventing many people from seeking VCT services. To promote effective HIV prevention programmes, significant efforts are needed to identify and remove all the fundamental causes of stigma and its associated discrimination against people known to be HIV-positive.

### Strengths and Limitations of the Study

This study had attempted to offer a number of insightful observations regarding the willingness and barriers to VCT among self-perceived healthy adults in resource-limited settings within the context of a generalised HIV epidemic.

The study had some limitations. The first important limitation the study relied on reported rather than actual VCT use. Future studies should consider prospectively collecting data which could include participants being presented with a card to present to the VCT centres within a specified period. However, this was beyond the scope of this study.

## CONCLUSION

Many barriers still exist that prevent people from taking up VCT services. We found that knowledge of VCT benefits was higher in urban areas; however, knowledge alone did not necessarily translate to a willingness to take up VCT services. Our study showed that various structural barriers are present, primarily stigma, but also health service constraints in terms of staff availability and competency and facility infrastructure. Increased efforts are needed to promote the up-take of VCT. Targeting health promotion efforts and further exploring the willingness of people to take up services in areas of low uptake would be beneficial.
